# Physical exercise, health, and disease treatment: The role of macrophages

**DOI:** 10.3389/fphys.2023.1061353

**Published:** 2023-04-25

**Authors:** Irineu O. M. Callegari, Guilherme Z. Rocha, Alexandre G. Oliveira

**Affiliations:** ^1^ Department of Physical Education, Bioscience Institute, São Paulo State University (UNESP), São Paulo, Brazil; ^2^ Department of Internal Medicine, State University of Campinas, Campinas, Brazil

**Keywords:** exercise training, inflammation, chronic diseases, metabolic diseases, immune response, innate immunity, health, active lifestyle

## Abstract

Subclinical inflammation is linked to comorbidities and risk factors, consolidating the diagnosis of chronic non-communicable diseases, such as insulin resistance, atherosclerosis, hepatic steatosis, and some types of cancer. In this context, the role of macrophages is highlighted as a marker of inflammation as well as for the high power of plasticity of these cells. Macrophages can be activated in a wide range between classical or proinflammatory, named M1, and alternative or anti-inflammatory, also known as M2 polarization. All nuances between M1 and M2 macrophages orchestrate the immune response by secreting different sets of chemokines, while M1 cells promote Th1 response, the M2 macrophages recruit Th2 and Tregs lymphocytes. In turn, physical exercise has been a faithful tool in combating the proinflammatory phenotype of macrophages. This review proposes to investigate the cellular and molecular mechanisms in which physical exercise can help control inflammation and infiltration of macrophages within the non-communicable diseases scope. During obesity progress, proinflammatory macrophages predominate in adipose tissue inflammation, which reduces insulin sensitivity until the development of type 2 diabetes, progression of atherosclerosis, and diagnosis of non-alcoholic fatty liver disease. In this case, physical activity restores the balance between the proinflammatory/anti-inflammatory macrophage ratio, reducing the level of meta-inflammation. In the case of cancer, the tumor microenvironment is compatible with a high level of hypoxia, which contributes to the advancement of the disease. However, exercise increases the level of oxygen supply, favoring macrophage polarization in favor of disease regression.

## Introduction

The epidemic of chronic non-communicable diseases (NCD) is rapidly growing due to the modern lifestyle, i.e., physical inactivity associated with the intake of high caloric diets, mainly composed of saturated fats and sucrose (Heindel et al., 2022). Among the NCD, the most prevalent are obesity, atherosclerosis, type 2 diabetes (T2D), and some cancers (Howe et al., 2013). NCD display a subclinical chronic inflammation, also known as meta-inflammation, that seems to have a pivotal role in the onset or worsening of these diseases. In this context, the role of macrophages is highlighted, since they act in the defense of the organism against pathogens, in tissue homeostasis, as well as markers of meta-inflammation and NCD (Varol et al., 2015). Macrophages are essential immune cells, that have high plasticity, and their phenotype varies according to the environment.

Didactically, macrophages have been classified in two extreme and opposing activations: classical or proinflammatory named as M1 macrophage and alternative or anti-inflammatory also known as M2 macrophage (Epelman et al., 2014; Murray et al., 2014; Geissmann and Mass, 2015; Varol et al., 2015). However, this dichotomy, by far, is not enough to characterize the high spectrum of existing phenotyping macrophage activation in humans (Vijay et al., 2020). Indeed, the M1/M2 polarization mode seems to fit to classify macrophage polarization only in vitro studies (Lauterbach and Wunderlich, 2017; Baek et al., 2020). Another possible macrophage designation is related to its origin, i.e., from the tissue as resident macrophage and monocyte-derived when it infiltrates into the tissue (Nahrendorf and Swirski, 2016). For this reason, in the current review we will adopt the following terminologies: M1-like or proinflammatory for macrophages that predominantly induce inflammation, and M2-like or anti-inflammatory for those that dampen inflammation or promote tissue repair.

**FIGURE 1:**
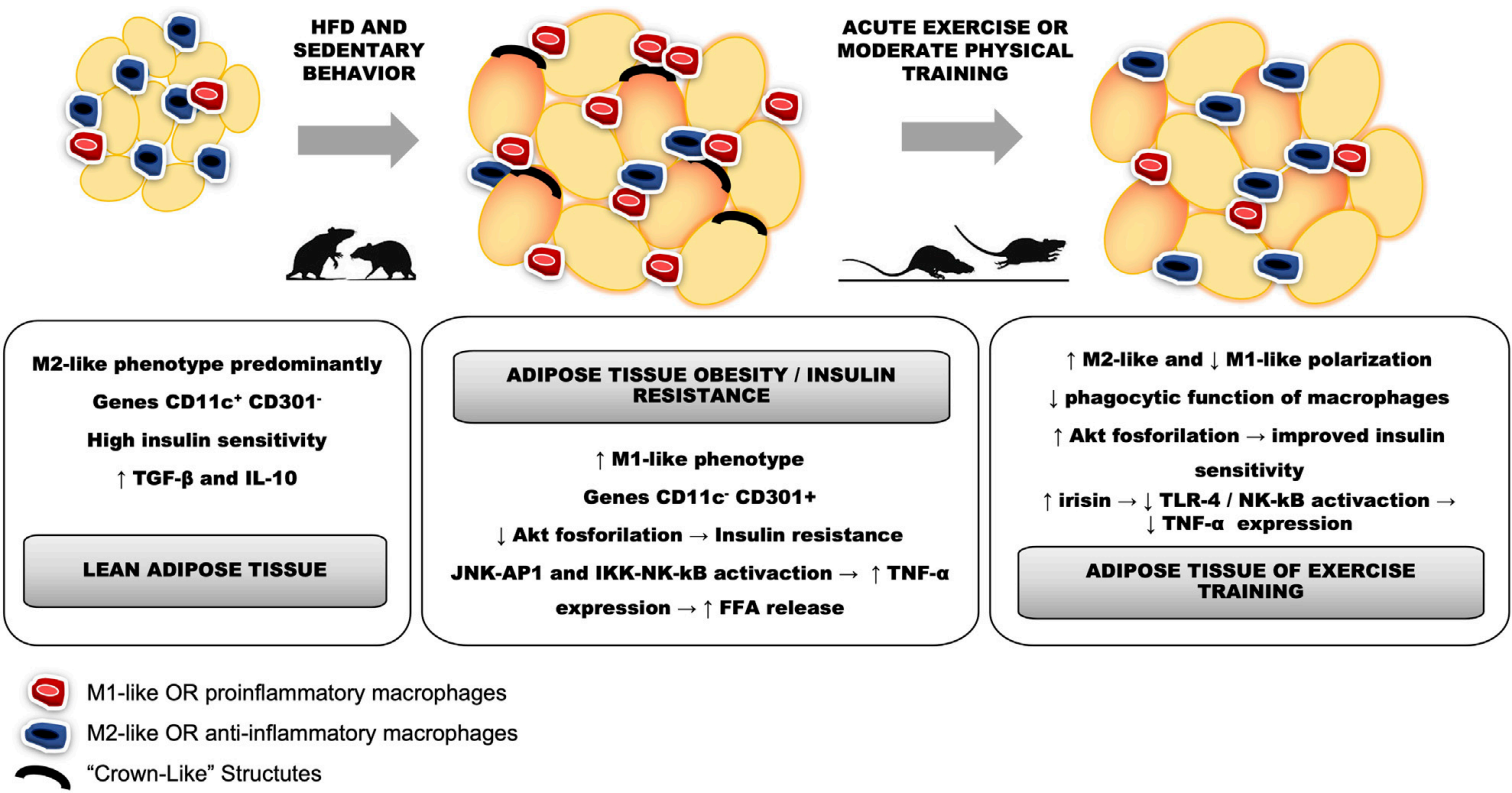
Differences between proinflammatory macrophages characterized by the M1-like phenotype, infiltrated and polarized in adipose tissue differently from the M2-like phenotype, represented by anti-inflammatory macrophages.

While M1-like phenotypes cells promote a proinflammatory Th1 response, the M2-like macrophages recruit Th2 and Tregs lymphocytes. In M1-like polarization, there is a predominance of the expression of TNF-α, IL-6, and IL-1β. This type of activation occurs mainly in the presence of lipopolysaccharides (LPS) and interferon-gamma (IFN-γ) (Parisi et al., 2018). Moreover, persistent macrophage activation towards M1-like can induce tissue damage, as occurs in chronic diseases (Sica et al., 2015). M2-like macrophage activation, mainly stimulated by IL-4 and IL-13, is characterized by higher IL-10 expression, homeostasis, and tissue repair (Rőszer, 2015). Thus, the ratio between the proinflammatory and the anti-inflammatory macrophage phenotypes, despite the existing limitations in relation to this dichotomy, has been used as a common model to understand meta-inflammation (Li et al., 2018) (Figure 1).

In contrast, it is accepted that physical exercise results in physiological alterations that can contribute to homeostasis and health. Indeed, the skeletal muscle (SM) contractions induce several responses in the whole body, including in the immune system (Krüger et al., 2016). During a single session of exercise, anti-inflammatory cytokines such as IL-1ra and IL-10 inhibit the production of the pro-inflammatory cytokine TNF-α, and alter the ratio between M1-like and M2-like polarization. Thus, regular physical exercise, considered a non-drug therapeutic strategy, induces protection against diseases and comorbidities due to its ability, among others, to potentiate the anti-inflammatory phenotype (Goh et al., 2016).

In this review, we highlight the behavior of macrophages on the effect that physical exercise plays in the treatment and prevention of NCD, due to the potential of exercise to control inflammation existing in metabolic disorders (Lancaster and Febbraio, 2014). Therefore, we start from the premise that physical exercise is a useful non-drug therapy for some diseases, especially with respect to its influence on macrophage infiltration and polarization.

## Obesity, insulin resistance and type 2 diabetes

Obesity, the most prevalent NCD in this century, is characterized by increased body weight and accumulation of comorbidities that increase the risk of mortality through multifactorial pathophysiology (Meister et al., 2022). Thus, the increase in white adipose tissue (WAT) inflammation during obesity has been associated with impaired insulin signaling, glucose intolerance, and changes in glucose and fatty acid metabolism (Antonopoulos and Tousoulis, 2017). While in lean adipose tissue macrophages (ATM) are primarily polarized towards the anti-inflammatory phenotype, during obesity onset there is a strong infiltration and polarization of M1-like macrophages into this human tissue (Kratz et al., 2014) according to Figure 1. Likewise, T2D is also characterized by chronic low-grade inflammation that exerts a pivotal role its pathophysiology (Donath and Shoelson, 2011). Indeed, T2D patients display elevated levels of proinflammatory cytokines such as TNF-a, CRP, IL-1β and IL-6 (Liu et al., 2016). In this context, regular physical exercise has emerged as an useful tool in the treatment of T2D since it has a prominent role in maintaining physiological levels of glycemia and its feature as an anti-inflammatory mediator (Abd El-Kader et al., 2015). Supporting this idea, studies demonstrated that physical exercise is able to boost the levels of anti-inflammatory cytokines IL-10 and IL-1receptor antagonist, that certainly help to promote an inflammatory environment (Lancaster and Febbraio, 2014). Also, Starkie and colleagues in a seminal study consistently demonstrated that 3 h of moderate cycling exercise resulted in an important reduction of TNF-a expression induced by endotoxin (Starkie et al., 2003).

**FIGURE 2:**
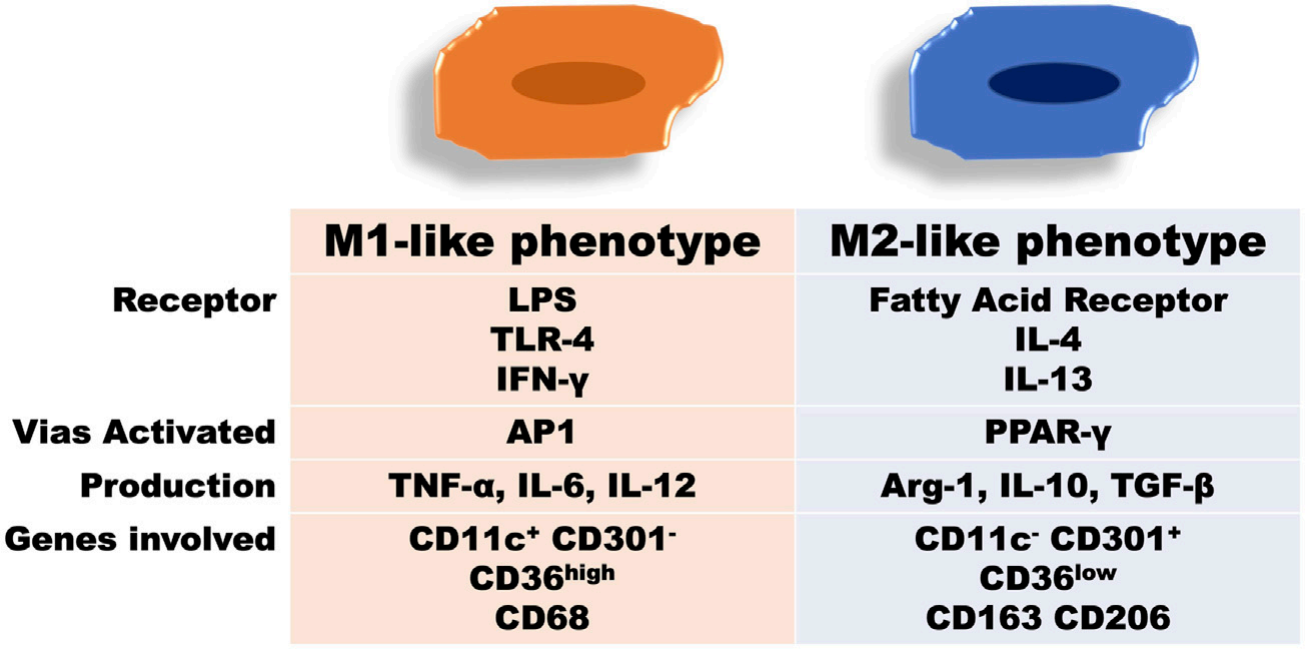
Changes in adipose tissue as a result of obesity development or physical exercise. A: lean adipose tissue from untrained rodents (controls) shows a predominance of the anti-inflammatory phenotype, whereby CD11c+ CD301-gene expression is related to increased levels of TGF-β and IL-10, consistent with normal levels of insulin sensitivity. B: hypertrophy in adipose tissue present in obesity leads to the formation of crown-like structures and alteration of the ratio M1-like and M2-like polarization, with proinflammatory predominance and CD11c- CD301+ gene expression compatible with the increase in TNF-α expression due to the activation of JNK-AP1 and IKK-NK pathways. This fact is directly related to the decrease in Akt phosphorylation in adipose tissue and the consequent presence of insulin resistance. C: acutely, exercise can restore the ratio M1-like and M2-like polarization while there is an increase in the M2-like phenotype and a decrease in the M1-like sense polarization, as well as its phagocytic function. Medium to long-term training can restore insulin sensitivity and Akt phosphorylation in adipose tissue, decreasing lipogenesis. Furthermore, increased irisin release from skeletal muscle may decrease TLR4/NK-kB activation and TNF-α expression.

In fact, obesity and IR have intracellular mechanisms linked to macrophage inflammation in WAT; for example, 4 weeks on a high fat diet (HFD) seem to be enough to induce glucose intolerance and impaired insulin signaling, as evidenced by reducing insulin-stimulated Akt phosphorylation levels, that correlate with an increase in macrophage infiltration and genes related to the M1-like phenotype (CD11c^+^) (Macpherson et al., 2015). In contrast, there is evidence that even a single session of exercise reduces proinflammatory polarization and circulating levels of MCP-1 and TNF-α in obese rats with IR (Oliveira et al., 2013). Likewise, acute aerobic exercise increases both adipose tissue and circulating levels of IL-10 in rodents, that certainly corroborate with anti-inflammatory macrophage polarization (Figure 2) (Macpherson et al., 2015; Zhu et al., 2015). Thus, there is inhibition of macrophage infiltration and upregulation of CD163 mRNA expression in the liver of obese C57BL/6 mice through moderate physical training (Jeong et al., 2015). Furthermore, in trained subjects the number of CD163 macrophages (M2-like phenotype) increases, while diet-induced weight loss tended to decrease CD68 genes (M1-like phenotype) in subcutaneous abdominal adipose tissue (SAT) (Fjeldborg et al., 2014). Contrary, moderate-intensity aerobic exercise did not show changes in insulin sensitivity and serum CD163 levels in human SAT, unlike what occurred in relation to caloric restriction (Fjeldborg et al., 2013).

In another molecular mechanism, mice with PPAR-γ gene deletion in macrophages are predisposed to developing obesity and IR through the increase of the proinflammatory gene expression of NF-κB (Odegaard and Chawla, 2011). In contrast, when PPAR-γ translocate to the cell nucleus, there is an increase in transcriptions of substances related to Th2 response with consequent polarization of anti-inflammatory macrophages (Lumeng et al., 2007; Silveira et al., 2016). This process is linked to the antagonistic activation between NF-κB and SIRT1, which stimulates the energy production from AMPK, PPAR-α, and PGC-1α to suppress inflammation (Kauppinen et al., 2013). Thus, inflammation increases activation of TNF-α receptor that induces the activation of the proinflammatory signaling pathways JNK/AP-1 and IKK-β/NF-κB in WAT (Andreani et al., 2007; Luo et al., 2020), which are compared to the combined effects of food restriction and moderate-intensity exercise in mice. Such a combination has greater additive effects on IR and obesity than the effect of exercise or caloric restriction alone. There was a reduction in M1-like polarization, identified by immunohistochemical analysis of CD86 and CD206, as well as a reduction in the secretion of the pro-inflammatory cytokines MCP-1 and TNF-α (Figure 2). Simultaneously, this treatment also promoted M2-like polarization of macrophages in the local WAT. Therefore, more evidence is needed comparing the isolated effect of each of the approaches compared to their combined effects during the management of obesity. Finally, CD11c+ (M1-like) and CD206+ (M2-like) cell subpopulations of the F4/80+ reveal that high-intensity exercise has a more pronounced efficiency, when compared to other training intensities, to reduce the M1-like phenotype and increase the M2-like phenotype in HFD-induced obese mice. In this case, the improvement in the inflammatory profile promoted by high-intensity training was accompanied by a greater reduction in TNF-a expression and an increase in the expression of the gene that encodes Arg-1 compared to lower training intensities (Pataky et al., 2019).

WAT stores free fat acids (FFA) after hypercaloric ingestion. However, FFA released adipose tissue (AT) to balance the energy state through lipogenesis and lipolysis. FFA binds to toll-like receptor-4 (TLR-4) and activates a proinflammatory response, resulting in the accumulation of ATM (Cullberg et al., 2014). Corroborating this, Kawanhisi and co-authors (Kawanishi et al., 2010) found that aerobic training on a treadmill for 16 weeks can induce macrophage polarization towards the M2-like phenotype in the WAT of obese animals. This exercise-induced macrophage polarization markedly inhibited the gene expression of TLR4 and TNF-α. Oliveira and colleagues (Oliveira et al., 2013) observed that even a single session of swimming exercise was enough to reduce activation of TLR-4 signaling pathway in HFD-induced obesity in rats. Interestingly, they found that such increase in M2-like polarization was mediated by reducing LPS circulating levels because when they administrated LPS immediately after acute exercise the macrophage polarization towards anti-inflammatory activation was blunted, in addition to increased gene expression of IL-4 and IL-10. It has since been postulated that physical exercise could increase the M2-like phenotype, thus attenuating inflammation in the WAT (Figure 2). The literature highlights that the therapeutic potential of physical exercise is due to the reduction in the recruitment and proliferation of immune cells in the WAT and decrease in the expression of inflammatory cytokines (Islam et al., 2021). Finally, oxidative stress has been highlighted in the treatment of obesity and IR since it was found that treadmill exercise reduces the gene expression of HIF-1α together with the mRNA of MCP-1, preventing the influx of macrophages into the epididymal AT of obese mice (Ye, 2008; Ahn and Kim, 2014). Such exercise effect is of particular interest as adipocyte hypertrophy results in a hypoxia environment, which stimulates the monocyte/macrophage chemotaxis into the WAT (Ye, 2009).

SM is the main organ by which insulin action lowers serum glucose levels (Furrer et al., 2017). Muscle contraction is one of the main mechanisms for improving insulin sensitivity, and recently its role has been associated with the behavior of macrophages in SM. There is an increase in the release of IL-6 and IL-15 due to the polarization of anti-inflammatory macrophages because of local muscle contraction (Ye, 2008; Ye, 2013). Corroborating with this, Reidy and colleagues (Reidy et al., 2018) investigated the differences in macrophage infiltration and polarization in SM among young and elderly adults after a rehabilitation program consisting of eccentric muscle contractions. Anti-inflammatory macrophages in human SM samples revealed a positive relationship with glucose uptake improvement due to physical exercise. However, resident macrophages of muscle tissue were not associated with changes in insulin sensitivity at the systemic level. For the authors, the polarization changes observed between the CD11b (M1-like) and CD163 (M2-like) genes may be associated with muscle growth and hypertrophy since such evidence was limited only to investigate the changes in SM. Tam et al. (Tam et al., 2012) evaluated the number of SM macrophages in young and older men after a program that combined aerobic and resistance exercises. They did not observe significant changes in the CD68 and CD206 macrophage mRNA, despite having found significant improvement in glycosylated hemoglobin (HbA1c).

In fact, physical exercise can lead to anti-inflammatory effects, if some intrinsic molecular aspects of WAT are achieved, such as high adipogenesis capacity, low extracellular matrix fibrosis, angiogenesis, beiging (brown colorization) of adipocytes and low infiltration rate of proinflammatory macrophages (Apostolopoulos et al., 2016). Thus, further studies are needed to better understand the effect of physical exercise on macrophage infiltration and polarization on SM tissue in individuals with T2D, especially if age and gender can affect these cells when exercising. Also, considering the current background, it is not clear whether changes in SM macrophages participate in the positive effect of physical exercise on insulin sensitivity in humans since, unlike what can be seen in the animal model (Pataky et al., 2019), there is no evidence in humans comparing the level of insulin sensitivity in SM with other endocrine tissues (Baek et al., 2020).

## Atherosclerosis

Recently, Pedersen and Shulman (Petersen and Shulman, 2018) claimed that meta-inflammation is the link between IR and atherosclerosis, because this condition leads to abnormalities in plasma levels of apolipoproteins (APO). Deficient mice in APO-E (APOE^-/-^ ) treated with a hypercholesterolemic diet show greater proliferation of macrophages located in the aortic root and arch (Baek et al., 2020). The formation of atheroma in the artery walls increases probably due to the accumulation of cholesterol resulting from low-density lipoprotein (LDL) in the subendothelial matrix, formation of lesions in arterial branches, hemodynamic strengthening of the endothelial cells, and the presence of nitric oxide synthase in the intimate tunic. Evidence in animal models of atherosclerotic regression showed that the balance between proinflammatory and anti-inflammatory macrophages in the plaques is dynamic, with a predominance of proinflammatory or M1-like in the progression of the disease and anti-inflammatory or M2-like showing a strong correlation with its regression (Peled and Fisher, 2014).

**FIGURE 3:**
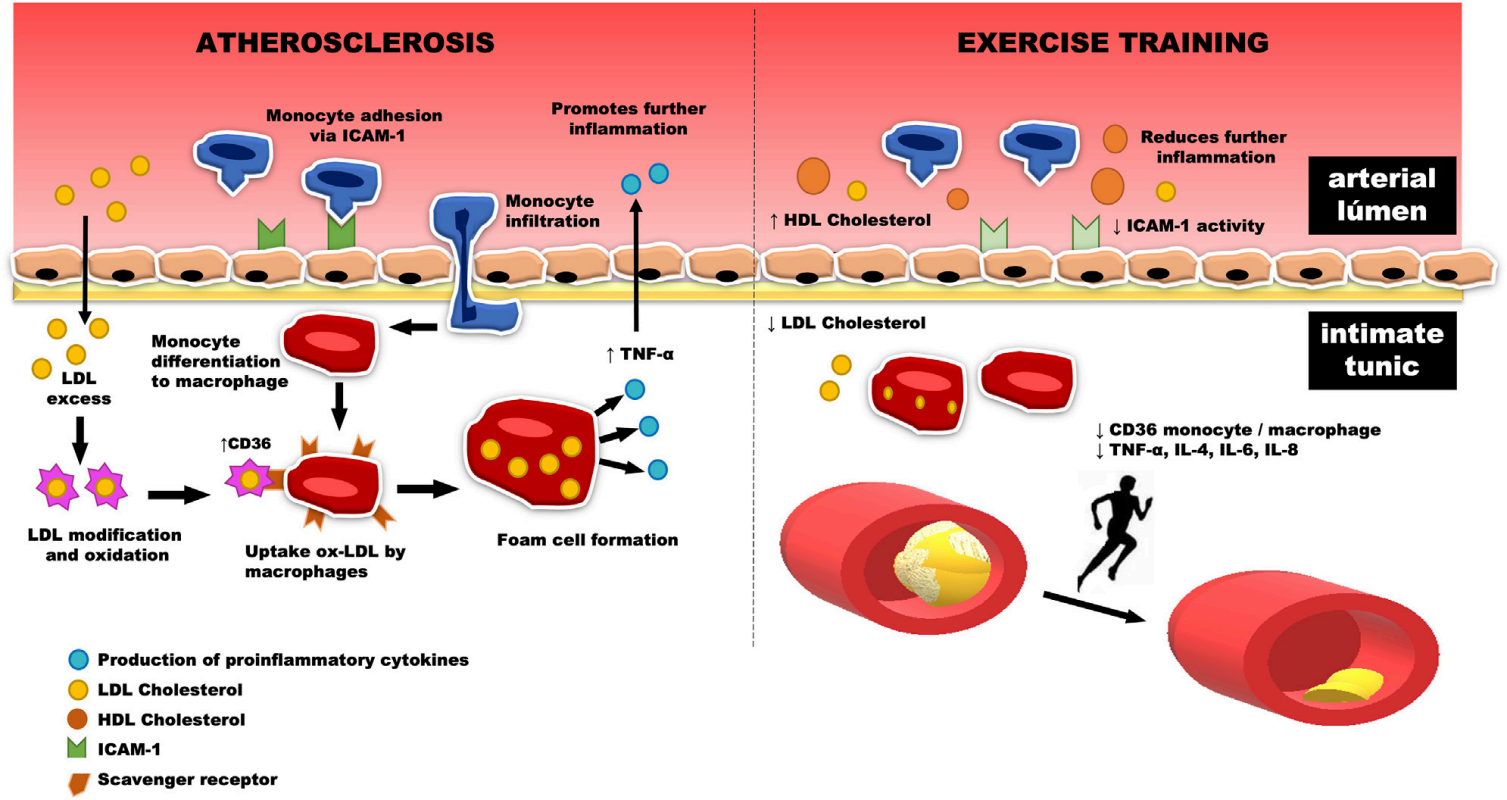
The increase in monocyte infiltration through the activation of the intercellular adhesion molecule-1 (ICAM-1), located in the vascular endothelium, promotes their differentiation to M1-like macrophages. The scavenger receptors of these macrophages bind to ox-LDL, increasing the release of the inflammatory marker CD36. In this way, the foam cell is formed, which in turn is related to the release of cytokines into the bloodstream, which induce an increase in the reverberative level of inflammation located in the aortic arch. This process can be, in part, reversed by physical exercise, which reduces the amount of LDL cholesterol and its consequent retention, a fact that decreases the amount of ox-LDL and the formation of foam cells in the intimate tunic, reducing the release of cytokines such as TNF-alpha, IL-4, IL-6, and IL-8 to the arterial lumen. Thus, there is a reduction in inflammation caused by the infiltration of monocytes and proinflammatory macrophages, followed by an increase in circulating HDL cholesterol levels. This fact leads us to believe that physical training is associated with a decrease in atherosclerosis plaques in the aortic arch.

In humans, it is well-established that moderate aerobic exercise is able to reduce the levels of plasma triglycerides (TG) and LDL, in addition to increase high-density lipoprotein (HDL) cholesterol (Søndergaard et al., 2014; Ferreira et al., 2017). In this context, there is evidence regarding the attenuation of atherosclerosis levels in individuals who underwent an 8-week walking program, accumulating 10,000 steps/day, three times a week (Yakeu et al., 2010; Winzer et al., 2018). However, no changes in the expression of monocytes CCR2^+^ and CD11b^+^ were observed through interventions based on reducing the sitting time of sedentary individuals (Yakeu et al., 2010; Noz et al., 2019). In contrast, Matsumoto and coworkers (Matsumoto et al., 2010) observed that aerobic exercise decreases in the recruitment of proinflammatory macrophages in the aortic arch after immunostaining, followed by a reduction in oxidized LDL (ox-LDL) expression in mice with HFD-induced dyslipidemia (Figure 3). Likewise, there was a significant reduction of proinflammatory macrophages and inflammatory markers, such as matrix metalloproteinase-3 in atherosclerotic lesions of trained mice fed on an HFD (Matsumoto et al., 2010; Kadoglou et al., 2013).

Wu and colleagues (Wu et al., 2019) recently investigated the anti-atherosclerotic effect of exercise in APO-E deficiencies in knockout c57bl/6 mice. After 8 weeks of treadmill training, the area of the transverse plate of the aortic tree was significantly reduced in the exercised groups, as well as the proinflammatory macrophage markers in histochemical and mRNA analyses. A deficiency of NPY receptor 1 was identified, which in turn implies a lower inflammatory state, verified by the inhibition of macrophage recruitment and the reduction of inflammatory cytokines (ICAM-1, TNF-α, IL-4, and IL -12 (Figure 3)). This finding is relevant, as it is similar to an experimental exercise protocol in which treatment with CD36 ligands, a type of scavenger receptor expressed in macrophages, was used to verify antiatherogenic effects in APOE^−/−^ mice, as well as the regulation of peripheral cholesterol trafficking. (Marleau et al., 2005).

Taken together, these data suggest that physical exercise ameliorates atherosclerosis, among other aspects, by reducing the infiltration of M1 macrophages and lipid content, leading to the belief that exercise is important in the regression of inflammation and treatment of dyslipidemia. However, since the data were obtained in animal studies, further clinical trials should be carried out to verify the relevancy of exercise to manage dyslipidemia in humans.

## Non-alcoholic steatohepatitis and non-alcoholic fatty liver disease

Non-alcoholic steatohepatitis (NASH) is considered an inflammatory condition in which excess fat is deposited in the liver (with or without the presence of fibrosis) and is considered one of the types of non-alcoholic fatty liver disease (NAFLD), which in turn represents the hepatic expression of metabolic syndrome (Manne et al., 2018). NASH is often associated with obesity and overweight, leading to the belief that strategies that combine dietary restructuring and aerobic exercise, with a reduction of more than 10% of body weight, can significantly improve disease progression (Higuera-de la Tijera and Servín-Caamaño, 2015). Interestingly, moderate exercise two to three times a week, without changing the diet or body weight, can also decrease hepatic steatosis in NAFLD after a period of 6–12 weeks in obese adolescents, thus highlighting the therapeutic role of physical exercise in this disease (van der Heijden et al., 2010). Indeed, the endurance exercise training is often recommended for NASH because it is able to reduce the excess of nutrients delivered to liver (Johnson et al., 2009; Brouwers et al., 2016).

In patients with NAFLD, a systematic review pointed out that aerobic exercise can induce lipolysis in the liver, activation of AMPK, UCP-1 and PPAR-γ, stimulation of GLUT4 translocation proteins, and an increase in adiponectin. In addition, compared to individuals with low NAFLD, exercise provides better control of glycemic levels due to higher glucose uptake in SM and a downregulation in hepatic gluconeogenesis (Hashida et al., 2017). Furthermore, vigorous-intensity exercise seems to be more effective for controlling NAFLD because it induces greater activation of hepatic AMPK and its downstream targets, such as restoring SIRT-1 expression in the liver (significantly decreased in a NAFLD model of HFD-fed rats) and leading to upregulation of target molecules associated with fatty acid oxidation and/or mitochondrial biogenesis (Cho et al., 2015). Conversely, a well-conducted randomized trial showed that high intensity and moderate intensity trainings with similar energy expenditure have similar outcomes for NAFLD patients. In addition, this study also demonstrated that the positive effects were achieved even without any alteration in body composition (Winn et al., 2018).

Rødgaard-Hansen et al. (Rødgaard-Hansen et al., 2017) evaluated 126 patients diagnosed with NAFLD who participated in a study with a proposed follow-up aiming at the practice of physical activity through individual counseling to complete 200 min/week of walking. After 3 months of intervention, the plasma inflammatory biomarker for macrophages scavenger CD163 showed a significant reduction, reaching healthy levels. However, there was no significant correlation between serum macrophages and measurements of total cholesterol and LDL, TG, fasting glucose, and total minutes of physical activity. Indeed, the results verified in an animal model have not yet been seen in humans, leading to the belief that interventions that explore different exercise intensities and volumes should be explored to better understand the role of physical exercise in the macrophage associated with hepatic markers in the context of NAFLD.

With respect to NASH, physical training seems to not induce any change in the plasma levels of the proinflammatory scavenger CD36 macrophage marker, however, it is also important to investigate the effects on liver (Butcher et al., 2008). In this context, the reversal of NASH through physical exercise seems to be related to macrophages’ polarization (Winzer et al., 2018). Thus, Kawanishii et al. (Kawanishi et al., 2018) sought to verify whether 16 weeks of physical training (60-min sessions, five times a week) would be able to decrease the proinflammatory expression of CD36 macrophages in the liver by analyzing the polarization towards M1-like through the CD11b^+^ and F4/80^+^ cells in mice. The results revealed that this macrophage phenotype, in addition to the protein expression of PPAR-γ and Nrf-2, was significantly higher in sedentary mice treated with HFD and water with a high fructose content than in exercised animals subjected to the same diet. These results suggest that physical training can improve liver inflammation and fibrosis by reducing the expression of CD36 in kupffer cells in the liver in NASH-induced mice.

In contrast, Henkel et al. (Henkel et al., 2019), observed that aerobic exercise increased the gene expression of the Adgre-1, a gene that encodes the expression of F4/80, in liver from *wistar* rats fed on HFD. Such result was accompanied by the higher protein expression of IL-1β, leading to the understanding that liver inflammation induced by diet can be aggravated by exercise. It should be noted that according to the authors, a high-intensity training protocol was not used, and that the increase in IL-1β expression was possibly HFD-dependent. However, running exercise on a treadmill can result in greater stress to the animals, thus contributing to these unexpected results. Since stress can disrupt the balance of intestinal microbiota, it is tempting to speculate that the increase in inflammation may also be related to changes in the intestinal microbiota. Thus, despite physical exercise being a well-accepted strategy in the treatment of liver disorders (Golabi et al., 2016), its role in liver inflammation levels, as well as the crosstalk between microbiota and liver still need to be more deeply investigated.

## Tumor microenvironment

Among many studies that reflect the importance of cancer prevention and treatment through physical exercise and given the complexity of the cellular demand imposed by such a disease (Howe et al., 2013), we limit ourselves to analyzing only macrophage behavior in this context. For this, we must understand that the tumor microenvironment (TME) can be interpreted by cell inoculation and cytotoxicity techniques in an experimental model. Often, such techniques lead to systemic responses antagonistic to the current understanding of the progression or reversal of the disease, leading to the belief that there are still many gaps to be filled for its complete understanding (Wright and Simone, 2016). Therefore, we will approach the subject given the influence that exercise has on macrophages in the inflammatory environment caused by cancer, since randomized studies indicate that chronic exercise can confer similar favorable effects in patients with primary cancer (Grande et al., 2014).

Pioneering evidence regarding cancer under the influence of aerobic training was given due to cell culture techniques, in which Jãpel et al. (Jäpel et al., 1992) measured the phagocytic activity of macrophages extracted from trained female NMRI mice. Before or after the intervention period, tumor cells, L-1210 or S-180, were inoculated, inducing leukemias and sarcolemma, respectively. On this occasion, macrophages from trained animals showed increased phagocytic activity, especially in the trained group before and after tumor inoculation (Himbert et al., 2017). Thus, it was postulated that exercise could demand a positive inflammatory response to cancer in rodents (Bacurau et al., 2000), even during high-intensity exercise (Bacurau et al., 2007), especially in senility (Lu et al., 1999), due to which not only the immune response but also the increase in vascularization linked to the oxygen supply would contribute to antitumorigenic effects (Hojman et al., 2018).

Corroborating this hypothesis, Almeida and colleagues (Almeida et al., 2009) verified in an animal model that, after 6 weeks of swimming training, the inoculation of tumor cells led to an increase in the infiltration of macrophages, which showed a correlation with the delay in the development of Ehrlich carcinomas. Likewise, treadmill exercise reduced the metastasis of lung tumors in mice by increasing the anti-tumor cytotoxicity of alveolar macrophages (Jones et al., 2009). Therefore, the increase in phagocytic activity as a result of physical exercise was related to the improvement of tumor cytotoxicity. The increase in the number of infiltrated macrophages reflects, in theory, the ability to attenuate growth or even regress the tumor (Ragan et al., 2013). However, studies in humans have not yet reached this same understanding, as it is likely that the side effects of chemotherapy make it impossible to carry out different training protocols.

In contrast, Keylock et al. (Keylock et al., 2008) stated that cancer would correlate with the inflammation inherent to aging since older people have chronic inflammation, harmful to wound healing, and related to the appearance of tumor growth factors. Thus, Ly and colleagues (Ly et al., 2010) observed a positive correlation between cancer progression and the polarization of macrophages towards M2-like since the expression of the F4/80, CD11c, PPAR-g, and CD163 genes are similar to the levels found for tissue repair, excess angiogenesis, hormonal decompensation, and immunosuppression caused by the progression of tumor factors and the development of metastases (Yang et al., 2019), as shown in Table 1. In addition, Ko et al. (Ko et al., 2014) found a significant decrease in the carcinoembryonic antigen of older women submitted to a 12-week program of aerobic exercises, combined with stretching and light-intensity strength exercises. Although the carcinoembryonic antigen is involved in monocyte and macrophage chemotaxis, the number of total leukocytes has not been altered (Goh et al., 2012). In this controversy, we understand that the comparison of cytotoxic effects of macrophages in the cancer microenvironment is dissociated from systemic inflammatory outcomes, especially concerning exercise.

**TABLE 1:** Differences between macrophage phenotype from primary tumor to cancer progression, metastases and the effects of physical exercise.

Tumor size	Accelerated tumor growth			Slows tumor growth	
Tumor-associated Macrophages (TAM) infiltration	M2-like predominantly	i M2-like (T quantitative MCP-1)	M2-like -+ M1-like M1-like predominantly	_s_l, M1-like i M2-like (t quantitative MCP-1)	M2-like predominantly (1 quantitative MCP-1)
Blood Flux	0_2_ deficiency	Hypoxia		Blood flow/02 supply	Angiogenesis
	Perfusion limited (presence of abnormal blood vessels)			Perfused vessels	
Oxigen Cells	1	II	III	T	TT
Response stimulus for macrophages	i HIF-la, IL-6 pSTAT-3	_s_l, VEGF, IL-10	i IFN-y, LPS	i L-arginine, NO, VEGF, MIP-la i PPAR-y	
Via Activaction	i TLR-4, NF-kB Th2 response		i TLR-4, NF-kB, ERK-1/2, JNK Th1 res onse p	1 TLR-4, NF-kB Th1 and Th2 response	1 TLR-4, NF-kB Th2 response
Cytokine production	,I, IL-12, TNF-a i TGF-13, IL-10		i IL-1, IL-12, ROS, TNF-a _s_l, TGF-r3, IL-10	,I, IL-12, TNF-a i TGF-13, IL-10	
Environment Effect	Tumor		Antitumor	Antitumor	

**FIGURE 4:**
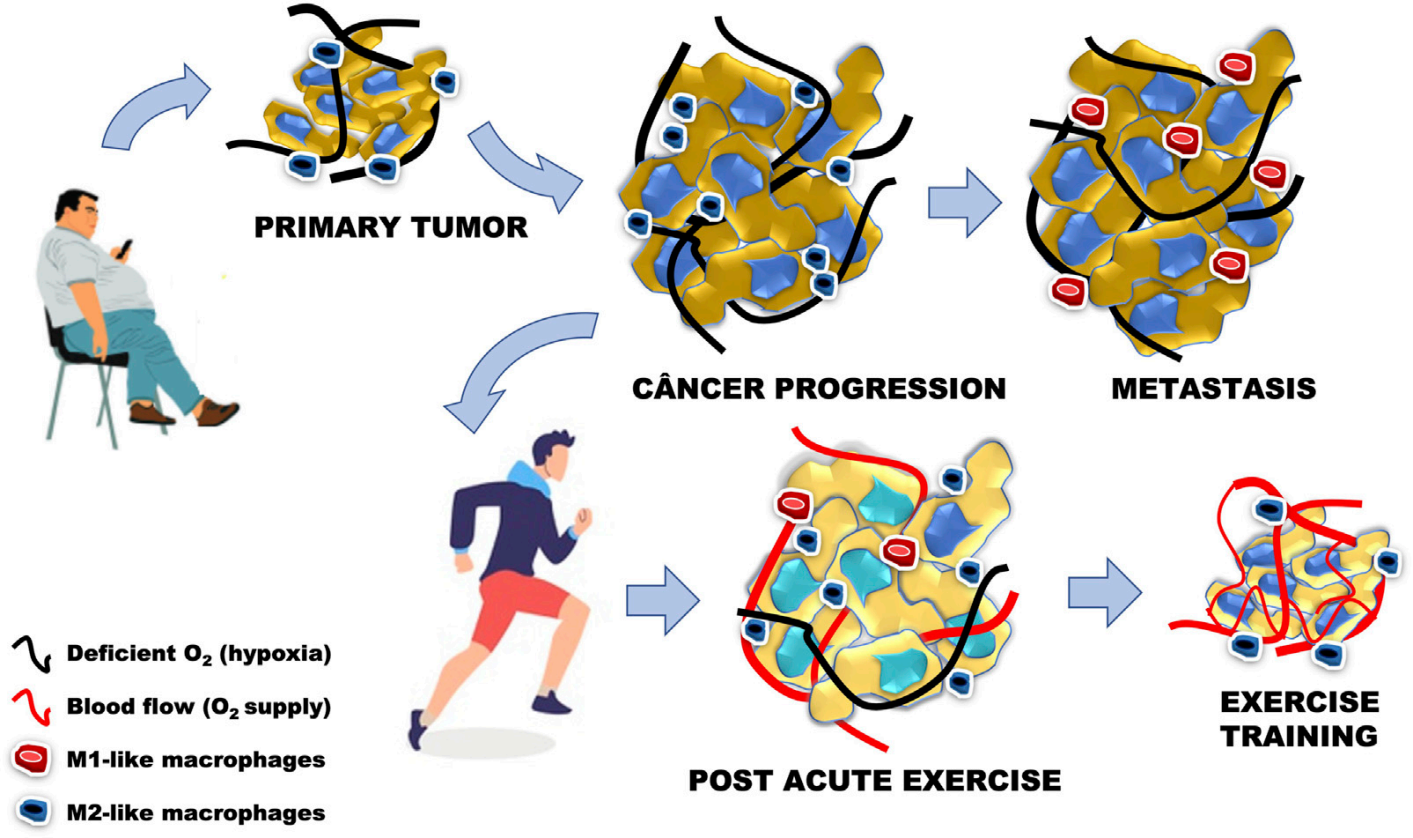
Cancer progression is related to the increase in cancer cells, M2-like phenotype and presence of hypoxia, similar to the primary tumor model related to sedentary behavior associated with the presence of obesity. In metastasis, the increase in the number of abnormal cells starts to present an M1-like phenotype, with maintenance of oxygen deficiency levels. Acute physical exercise stimulates local and systemic angiogenesis, leading to a greater supply of oxygen to cancer cells. The adaptations generated by low-intensity aerobic physical training led us to believe that the increase in the anti-inflammatory phenotype in the tumor microenvironment may decrease the number of abnormal cells, while increasing the number of blood vessels and oxygen supply.

It seems that the alternative polarization achieved in the context of exercise used as non-drug therapy, favors wound healing and tissue repair, as seen in the TME, both contrary to the proinflammatory profile mentioned earlier in this review. However, there is evidence that the LPS stimulus induces the proinflammatory polarization of M1-like macrophages in an animal model by activating the NF-kB, ERK-1/2, and JNK signaling pathways, similar to what is seen in the presence of metastases (Hawley et al., 2014). In turn, such cascades of inflammation were reduced by light aerobic exercise, even in the exercised group that received treatment with LPS during the intervention period (Shi et al., 2020). In agreement, Goh and Ladiges (Goh and Ladiges, 2014) observed in rodents that running on the treadmill increases the expression of anti-inflammatory markers (CD163) due to reduced inflammation related to the pre-development of cancer and by the decrease of the expression of M1-like markers (CD11c and TLR4). Conversely, Abdalla and collaborators (Abdalla et al., 2013) concluded that physical activity promotes a standard profile of anti-tumor Th1 response when analyzing the polarization of immune cells (Figure 4). Such a discrepancy in the effect of physical exercise on the polarization of macrophages in the TME may result from differences in the experimental protocol regarding the type of exercise or even its intensity and volume (Yang et al., 2019).

Many studies have associated cancer incidence with the obesity pandemic due to the close inflammatory relationship built between both (Kang et al., 2017; López-Janeiro et al., 2020). Obesity is one of the main modifiable contributors to cancer mortality, as the subclinical inflammation in WAT stimulates tumor progression. Besides, tumor-associated macrophages (TAM) induce systemic inflammation during obesity, similar to the M1 polarization of proinflammatory ATM during obesity (Springer et al., 2019). As already discussed previously, physical exercise has a fundamental role in controlling inflammation in WAT, mainly due to the attenuation of the classic inflammatory phenotype and increased M2-like polarization. In this context, although the protection mechanism induced by exercise is not clear, TAM are among the targets of the responses that physical exercise achieves in AT (Goh et al., 2014), largely due to the transition from M1-like to M2-like phenotype in acutely exercised rodents and humans (Kerr et al., 2017).

In the context of this relationship between cancer and ATM, Dieli-Conwright et al. (Dieli-Conwright et al., 2018) submitted obese postmenopausal women surviving breast cancer to an aerobic exercise protocol combined with resistance exercise for 16 weeks. The exercises were supervised three times a week, and the participants provided fasting blood samples and superficial subcutaneous biopsies of abdominal AT at the beginning and after the intervention period. There was attenuation of the pro-inflammatory phenotype combined with an increase in anti-inflammatory markers in AT and a significant reduction in systemic inflammation and body composition. TAM represent the highest proportion of leukocytes present in this type of cancer, thus, they are an attractive target for immunotherapy (Himbert et al., 2017). The scarcity of studies prevents us from stating that exercise acts directly in the treatment of breast cancer; however, data such as the presented in this review lead to the understanding that exercise may have an influence on decreasing the side effects of the disease, such as cachexia and increased AT inflammation (Kerr et al., 2017).

When faced with epidemiological evidence linked to specific types of tumors, such as breast cancer, for example, exercise seems to act in the progression of inflammation present in the disease (Meneses-Echávez et al., 2016). BALB/C mice with an inducible invasive breast cancer model, employing the 4T1 syngeneic tumor cells, showed a significant reduction in the macrophage and CD34 (vascular endothelial cell marker) marking during the tumor progression phase in the groups that covered higher distances from running on a treadmill (Pettan-Brewer et al., 2014). Breast cancer patients demonstrate a higher concentration of CD14^+^ CD16^+^ monocytes in the blood, correlated with the size and initial growth of the tumor. Thus, an intervention through aerobic training combined with resistance exercises ensured a significant decrease in this class of monocytes, as well as decreased production of endotoxin-stimulated TNF-α *in vitro* (Gonzalez-Carmona et al., 2008). However, despite this study demonstrating the role of exercise in attenuating inflammation through the reduction of circulating immune cells, to date, we are not aware of evidence that has addressed the prescription of physical exercise in modulating the infiltration and polarization of macrophages in tumors.

Regarding tumors affecting the gastrointestinal tract, McClellan et al. (McClellan et al., 2014) examined the effects of 12 weeks of treadmill exercise on markers associated with macrophages in mice with a mutation that leads to the formation of intestinal adenocarcinomas. Samples of intestinal polyps revealed that physical training led to a significant decrease in the expression of mRNA of the F4/80 gene and the M2-like polarization markers (CD206, CCL22, and Arg-1), followed by the reduction of IL-12 gene expression, a marker associated with the proinflammatory M1-like phenotype. Thus, the intervention was sufficient to recruit T lymphocytes and reduce the progression of colon cancer. In a subsequent study, interval running exercise on a treadmill associated with doxorubicin treatment, a drug that has the side effect of losing SM, resulted in the selective depletion of proinflammatory macrophages (CD68+) in the acute phase of treatment in muscle tissue, a fact that did not occur with anti-inflammatory macrophages (CD163+) (Huang et al., 2017). Given that macrophages are the most abundant immune population in the TME related to colorectal cancer (Bader et al., 2018), the development of new strategies that combine the anti-inflammatory effect of exercise with pharmacological therapy seems to be linked to the reduction of the deleterious effects of the disease and immunosuppression, despite the scarcity of works on humans. Thus, it becomes evident the need to conduct more studies in humans with different protocols to elucidate the effects of exercise in controlling inflammation mediated by macrophages and other immune cells during cancer treatment, whether in their microenvironment or their systemic effects.

## Final considerations

Increased macrophage polarization and infiltration towards proinflammatory phenotype reveals an inflammatory disorder due to HFD-induced obesity and an important risk factor for the development of T2D and atherosclerosis. The presence of macrophages in WAT and increased gene expression of CD11c+ and CD301- indicates polarization towards proinflammatory phenotype, while the anti-inflammatory phenotype is characterized by the expression of CD11c− and CD301+. The dynamism of the proinflammatory/anti-inflammatory phenotypes shows that the increase in the proinflammatory macrophage phenotype is related to glucose intolerance, IR, and progression of the size of atherosclerotic plaques.

In contrast, aerobic exercise restores Akt phosphorylation and leads to an increase in IL-10 expression, indicating an increase in anti-inflammatory macrophages. In trained individuals, the number of CD163 macrophages in the SAT, WAT, and liver increases, while diet-induced weight loss tends to decrease CD68 genes (M1-like phenotype) and LPS levels. In SM, being the main organ related to glucose metabolism independent of insulin action, CD68 and CD206 macrophages showed no changes in mRNA analysis, but the CD11c and CD206 phenotype shows that high-intensity exercise has greater efficiency to reduce the M1-like phenotype and increase the M2-like phenotype.

Surprisingly, in the studies referring to NAFLD/NASH there was no significant correlation between the change in the phenotype of serum macrophages and the measurements of total cholesterol, LDL, TG, fasting glucose and total minutes of physical activity for the experimental models discussed in the present work. We noticed that the improvement of M1-like markers (scavenger CD163, CD36, CD11b^+^ and F4/80^+^) occurred more significantly, and only two proposals revealed a significant effect of exercise on NAFLD. First, the combination of dietary re-education and aerobic exercises capable of reducing more than 10% of body weight were enough to improve the progression of the disease. Second, high-intensity aerobic exercise was the closest to metabolic regulation and mitochondrial biogenesis, verified through the activation of hepatic AMPK and its downstream targets.

The relationship between TME and macrophage plasticity presents a different starting point from the regular understanding discussed in this work, and the controversies on the subject are based on the different approaches to the treatment of the different types of cancer that exist, as well as the experimental design pattern identifying different responses in relation to the pattern of understanding about TME and macrophage infiltration at the tissue or systemic level. However, the influence that exercise exerts on macrophages in the inflammatory environment caused by cancer indicates that chronic exercise confers favorable effects similar to the other diseases discussed in this work. Despite the advance of cancer having a TME characterized by the anti-inflammatory phenotype, the increase in the expression of these markers *in vitro* (CD206, F4/80, CD163, CD11c) together with the increase in the number of macrophages infiltrated in the tissue exposed to the primary tumor reflects, in thesis, the ability to attenuate the growth or even regress the tumor. However, human studies have yet to reach this same denominator to date.

In general, this review showed that the polarization between anti-inflammatory and pro-inflammatory macrophages is highly dynamic, which has certainly impacted the understanding of physiological stimuli that modulate macrophage activation and tissue functions in the context of physical exercise.
